# The impact of mindfulness on working memory-related brain activation in breast cancer survivors with cognitive complaints

**DOI:** 10.1007/s11764-023-01484-0

**Published:** 2023-11-03

**Authors:** Michelle Melis, Jeroen Blommaert, Katleen Van der Gucht, Ann Smeets, Brenna C. McDonald, Stefan Sunaert, Andra Smith, Sabine Deprez

**Affiliations:** 1 Department of Imaging and Pathology, Translational MRI, KU Leuven, Herestraat 49—box 7003, 3000 Leuven, UZ Belgium; 2 Research Foundation Flanders (FWO), Flanders, Belgium; 3 Leuven Cancer Institute, KU Leuven, Leuven, Belgium; 4 Leuven Brain Institute, KU Leuven, Leuven, Belgium; 5 Department of Oncology, KU Leuven, Leuven, Belgium; 6 Leuven Mindfulness Centre, Faculty of Psychology and Educational Sciences, KU Leuven, Leuven, Belgium; 7 Department of Rehabilitation Sciences, Neuromodulation Laboratory, Biomedical Sciences Group, KU Leuven, Leuven, Belgium; 8 Tilburg School of Social and Behavioral Sciences, Tilburg University, Tilburg, The Netherlands; 9 Multidisciplinary Breast Center, Department of Surgical Oncology, University Hospitals Leuven, Leuven, Belgium; 10 Department of Radiology and Imaging Sciences, Indiana University School of Medicine and Indiana University Melvin and Bren Simon Comprehensive Cancer Center, Indianapolis, IN USA; 11 Department of Radiology, University Hospitals Leuven, Leuven, Belgium; 12 School of Psychology, University of Ottawa, Ottawa, Canada

**Keywords:** Breast cancer, Mindfulness, Cognition, fMRI, Working memory, Task based

## Abstract

**Purpose:**

Cancer-related cognitive impairment (CRCI) has been associated with altered brain activation after chemotherapy in areas related to working memory. Hence, improving working memory capacity and associated brain activation might aid in the recovery of CRCI. In this study, we investigated the potential of a mindfulness-based intervention (MBI) to impact working memory-related brain activation.

**Methods:**

Female breast cancer survivors reporting cognitive complaints (*N*=117) were randomized into a mindfulness (*n*=43; MBI), physical training (*n*=36; PT), or waitlist control condition (*n*=38; WL). Participants completed MRI scans before the intervention, immediately after, and three months post-intervention. Task-based functional MRI was used to measure differences between groups over time in working memory-related brain activation while performing a visual-verbal n-back task.

**Results:**

Data of 83 participants (32/26/25 MBI/PT/WL) was included. Compared to the waitlist group, MBI participants showed reduced task-related activation in the right middle frontal and angular gyrus and increased activation in the right dorsal posterior cingulate cortex over time. Compared to the physical training group, MBI participants showed reduced brain activation in the bilateral superior parietal lobule and right dorsal anterior cingulate cortex over time. No differences between physical training and no intervention were identified.

**Conclusion:**

This study showed that an 8-week mindfulness-based intervention can significantly alter brain activation across brain regions involved in working memory, attentional control, and emotion processing during performance of a working memory task. This might aid in the recovery of CRCI.

**Implications for Cancer Survivors:**

Mindfulness might alter brain activation patterns while performing a working memory task, which might ultimately aid in restoring higher order cognitive functions.

## Introduction

After treatment, one out of four breast cancer survivors shows impaired cognitive functioning [1]. This is known as cancer-related cognitive impairment (CRCI) and includes impaired attention, memory, executive function, and processing speed. As CRCI can have a long-lasting impact on survivors’ quality of life, most breast cancer survivors wish to receive support [2]. While CRCI has become an important area of research, more research on interventions targeting CRCI is needed [3].

Ideal interventions for CRCI should impact the different pathophysiological mechanisms of CRCI, as various mechanisms have been proposed that could alter cognition, brain structure, and function [4, 5]. For example, using task-based fMRI, reduced working memory-related brain activation in the frontal cortex has been observed immediately after chemotherapy. These findings suggest a decreased ability to compensate for functional impairment caused by cancer treatment [6, 7]. In contrast, one year after chemotherapy treatment, frontoparietal brain areas might show hyperactivation. This hyperactivation suggests that, although breast cancer survivors regain their ability to perform within normal ranges on working memory tasks, they need more resources to maintain the same performance level than before treatment [6]. Hence, interventions that can reduce the need for additional resources (potentially leading to fatigue), and thus reduce hyperactivation in frontoparietal areas, might aid in the recovery of CRCI.

A potential candidate for reducing CRCI is a mindfulness-based intervention (MBI). MBIs are psychological interventions, designed to train individuals to pay attention to present-moment experiences in a non-judgmental way [8]. It has previously been reported that standardized MBIs might improve working memory capacity in clinical and non-clinical samples [9, 10]. MBIs might train working memory capacity by increasing the awareness of information that is being processed and manipulated in working memory [11]. However, little is known about the underlying neural correlates [12]. To the best of our knowledge, no studies have been conducted in breast cancer survivors assessing the impact of MBI on working-memory related brain activation.

Therefore, in this longitudinal randomized controlled trial (RCT), we used fMRI to investigate the impact of MBI on working-memory related brain activation in breast cancer survivors with cognitive complaints, compared to active (physical training) and waitlist control groups. We hypothesized that both MBI and physical training would alter working memory-related brain activation compared to no intervention, but that the reduction would be larger after MBI.

## Materials and methods

The study protocol has been described in more detail elsewhere [13]. 

### Participants

Recruitment took place at University Hospitals Leuven and via flyers on social media. Study eligibility was determined using medical records. Interested candidates received the informed consent form and the Cognitive Failures Questionnaire (CFQ). Eligible participants were 18–65 years old, diagnosed with breast cancer with or without solitary metastases (except solitary brain metastases), received chemotherapy 6–60 months before enrolment, and were native Dutch speakers. Participants were excluded if they had MRI contraindications, previously received meditation training, or were diagnosed with intellectual disability, neurologic, or psychiatric disorders. Only participants with significant cognitive complaints (CFQ total score >42.9 (mean+1SD study Ponds et al., 2006 [14]) or at least two of the four extra CFQ questions > mean+1SD study Ponds et al., 2006 [14]) were included.

### Design and study procedure

The study was registered at ClinicalTrials.gov (NCT03736460). Participants were randomized across three groups: mindfulness, physical training, or waitlist control. The random number generator MinimPy (http://minimpy.sourceforge.net/) was used for randomization by an independent researcher. Stratification was based on time since chemotherapy, age, and endocrine therapy. Researchers were blinded to participants’ group allocation.

Neuropsychological tests and questionnaires, MRI of the brain, and blood samples were collected. The findings regarding the neuropsychological tests and questionnaires are described elsewhere [15], as well as the structural MRI [16] and resting state fMRI and biomarker results [17]. All participants were assessed before the intervention (t1), after the intervention (t2), and three months post-intervention (t3). After finishing all assessments, participants in both control groups could receive the MBI.

### Interventions

#### Mindfulness-based intervention

The intervention was based on Mindfulness-Based Stress Reduction [18] and Mindfulness-Based Cognitive Therapy for patients with cancer [19]. The eight-week program consisted of four 3-hour group sessions, once every two weeks, with the option of in-between online support from the trainer. Participants had to practice daily at home with audio recordings. Sessions included guided experiential mindfulness exercises (e.g., focus on the breath, bodyscan, breathing space, mindful yoga, insight and walking meditation) [13], sharing experiences, reflection, psychoeducation, and review of home practices. Two certified mindfulness trainers experienced in oncology provided the training. Attendance and the amount of home practice were documented.

#### Physical training

The physical training program was based on the recommended levels of physical activity for adults [20] and the existing breast cancer rehabilitation program at University Hospitals Leuven. Four 2-hour group sessions were spread over eight weeks. Sessions included psychoeducation, endurance and resistance training, stretching, balance and relaxation exercises, sharing experiences, and reviewing homework. Participants were asked to train endurance at home for 150 minutes a week and resistance for 2–3 times a week [20]. A physiotherapist experienced in oncology rehabilitation provided the training. Attendance and the amount of home practice were documented.

### MRI acquisition

Scans were acquired on a 3T Philips Achieva scanner with a 32-channel phased-array head coil. We collected 3D-TFE T1-weighted images (voxel size=0.8×0.8×0.8mm^3^, TR/TE=5.8/2.6ms, FOV=256×240×166mm^3^). Task-based functional images were acquired with a whole-brain T2*-weighted echo-planar imaging sequence (voxel size=2.14×2.14×3.30mm^3^, TR/TE=1500/33ms, FOV=240×240×139mm^3^, flip angle=80°, 280 dynamic scans in ~7min). Prior to processing, all images were converted to the Brain Imaging Data Structure [21] and visually inspected for artefacts.

### Working memory task

We used a visual-verbal n-back task to assess working memory-related brain activation, adopted from McAllister et al. [6, 22]. During scanning, participants watched a screen showing consonant letters presented one every three seconds. A blocked design was used to present 0-, 1-, 2-, and 3-back conditions. Each block lasted 27 seconds with a three-second instruction. Participants used a button press device to indicate whether the current letter was the same as the letter presented 0, 1, 2, or 3 items before. The four conditions were presented three times in pseudorandom order (12 blocks, 7.09 minutes). Participants rehearsed a version of the task before scanning to ensure they understood the task correctly. Stimuli were presented and programmed using Presentation (Neurobehavioral Systems), while recording response accuracy (true positive and false positive responses) and reaction times (in seconds). The accuracy percentage for each condition was calculated, correcting for guessing:

$$Accuracy\ \left[\%\right]=\left(\frac{True\ Positives}{7}-\frac{False\ Positives}{20}\right)\times 100$$

[6]. Analysis of variance (ANOVA) was used to test for baseline differences between the groups. If the accuracy percentages and reaction times differed between the groups at baseline, the parameters were included as a covariate in the second level between-group analysis. Additionally, if a participant scored less than 60% correct on both the 0-back and 1-back condition, the participant was assumed to not properly engage in the task and excluded from the analysis.

To test if performance changed within the groups over time, we used two-level linear mixed models with a random intercept (participant) and with group and time-point as fixed effects in R version 4.0.3 (lme4) [23]. All values were scaled so that the standardized coefficients provide information about the effect size [24]. We corrected for multiple comparisons with the Benjamini-Hochberg procedure [25] by adjusting the *p* values for the two performance measures for every task (i.e., accuracy percentage and reaction time for 0-, 1-, 2-, and 3-back) (*n*=2).

### Task-based fMRI preprocessing and analysis

Visual inspection of the images was performed to ensure data quality. Additionally, quantitative quality measures were computed using mriqc 21.0.0rc2 [26]. The data identified as lowest quality, based on visual inspection and quantitative metrics, were checked for exclusion by a neuroradiologist (SS). Preprocessing was performed using fMRIPrep 20.2.7 and included realignment, coregistration, slice timing correction, normalization to MNI space (MNI152NLin2009cAsym), tissue segmentation, and automated motion correction using independent component analysis (ICA-AROMA). Smoothing was completed using SPM12 with FWHM=6mm [27].

To investigate longitudinal group differences in task-related brain activation, we performed a general linear model analysis in SPM12 [27], using six motion parameters and global signal as regressors. Contrast images comparing task conditions were created for each participant for second level between-group analysis. A flexible factorial design was used with group-by-time and subject factors to investigate group-by-time interaction effects. We compared brain activation changes between the mindfulness and the two control groups, as well as between the physical training and waitlist group. Analyses focused on the most challenging working memory load conditions (2back-0back and 3back-0back), based on previous research showing that these contrasts are most sensitive to group differences [28, 29]. Significant *p* values were family-wise-error corrected on cluster level at *p*<.05, with an uncorrected voxel-level threshold of *p*<.001.

If a significant cluster was identified, we performed post hoc analyses to gain insight in the direction of the group-by-time interaction effects. Mean values for clusters showing significantly altered activation from baseline to post-intervention were extracted using the MarsBar toolbox in SPM12 for every participant/session. Within these clusters, we calculated changes in brain activation within the groups over time using linear mixed models with a random intercept (participant) and with group and time-point as fixed in R version 4.0.3 (lme4) [23]. Results were considered significant at *p*<.05.

### Correlation with self-report questionnaires and task performance

We investigated the association between significant changes in brain activation and (1) changes on self-report questionnaires assessing subjective cognitive function, emotional well-being, fatigue, quality of life, and mindfulness skills, and (2) task performance. More details about the five questionnaires used as part of this larger RCT can be found elsewhere [15]. First, we calculated change scores (t2−t1 and t3−t1) of the mean values of the significant clusters and (1) of the scores on each questionnaire, and (2) accuracy percentage and reaction time on the 2-back and 3-back condition per participant. Second, we calculated the Spearman correlation between the change in brain activation and the change in questionnaire scores/task performance across all groups. Results were considered significant at *p*<.05. The Benjamini-Hochberg procedure was used to correct for multiple comparisons for (1) each of the five questionnaires within a significant brain region (*n=*5) or (2) the two measures of task performance (i.e., accuracy percentage and reaction time) (*n*=2) [25].

## Results

### Enrolment and attrition

**Fig. 1:**
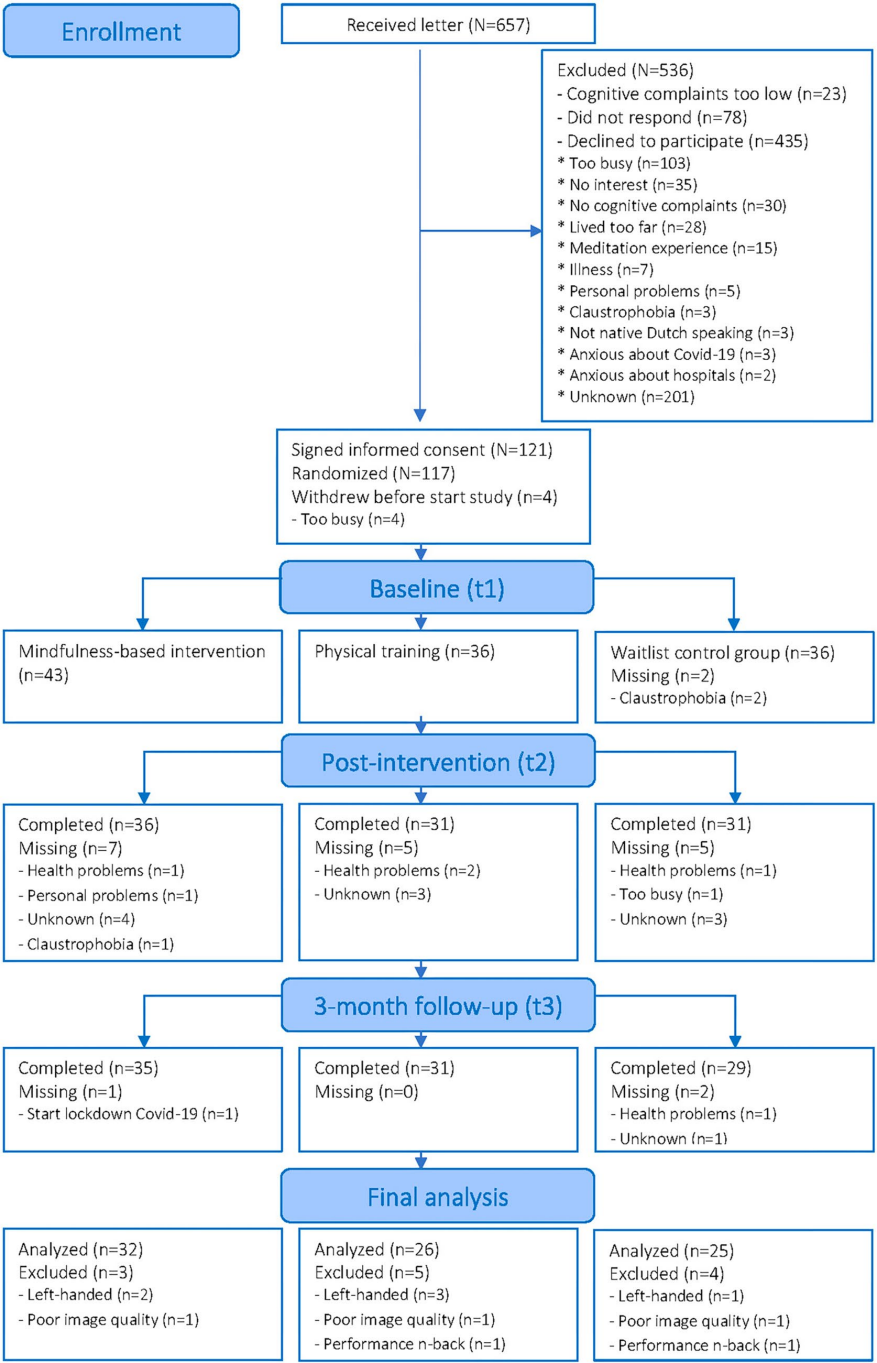
CONSORT flow diagram increase font in boxes

Of the 657 potentially eligible participants, 78 did not respond, and 435 declined to participate. Of the 144 candidates that were interested, 23 were excluded because they scored below the cutoff for cognitive complaints on the CFQ (for more details, see Fig. 1). The informed consent was signed by 121 breast cancer survivors with cognitive complaints. Before the baseline measure, four participants dropped out because of a lack of time. Therefore, 117 participants were allocated to a mindfulness (*n*=43), physical training (*n*=36), or waitlist control condition (*n*=38). Of these participants, 86.1% attended at least 3 sessions of the MBI, and 80.5% attended at least 3 physical training sessions. In total, we collected complete task-based fMRI data of 95 participants. Data of six participants were excluded because they were left-handed, two were excluded because they performed less than 60% on both 0- and 1-back, and data quality of three participants was classified as too low, resulting in data of 83 participants (MBI=32; PT=26; WL=25) included in the final analysis (Fig. 1). Detailed information about the amount of home practice reported by the included participants can be found in Table 1.

**Table 1:** Amount of home practice reported by included participants after the intervention

Home practice *N* (%)	Post-intervention (t2)		Three-month follow-up (t3)	
	Mindfulness	Physical training	Mindfulness	Physical training
Never	0 (0.0)	0 (0.0)	0 (0.0)	1 (3.8)
Less than once a month	0 (0.0)	0 (0.0)	2 (6.3)	0 (0.0)
About once a month	0 (0.0)	0 (0.0)	1 (3.1)	0 (0.0)
Several times a month	1 (3.1)	2 (7.7)	4 (12.5)	0 (0.0)
About once a week	6 (18.7)	1 (3.8)	8 (25.0)	1 (3.8)
Several times a week	15 (46.9)	15 (57.7)	15 (46.9)	10 (38.5)
Daily	7 (21.9)	0 (0.0)	1 (3.1)	0 (0.0)
Not reported	3 (9.4)	8 (30.8)	1 (3.1)	14 (53.8)

### Participant characteristics

Groups were comparable at baseline on demographic and treatment variables (Table 2). All women were 28–63 years old (mean=48.6, SD=8.1), and the average time since chemotherapy completion was 26 months (SD=14.9).

**Table 2:** Demographic and medical characteristics of included participants at baseline

	Mindfulness (*n*=32)		Physical training (*n*=26)		Waitlist (*n*=25)	
	Mean (SD) or *N* (%)	95% CI	Mean (SD) or *N* (%)	95% CI	Mean (SD) or *N* (%)	95% CI
Age (years)	47.8 (7.7)	[44.9, 50.7]	49.0 (7.4)	[41.9, 56.2]	49.3 (9.5)	[42.0, 56.5]
Verbal IQ	109.7 (6.1)	[107.6, 111.7]	111.2 (5.8)	[106.1, 116.4]	106.9 (5.6)	[101.8, 112.1]
Months since chemotherapy	27.1 (15.6)	[21.8, 32.3]	22.0 (13.1)	[8.9, 35.1]	27.3 (15.9)	[14.1, 40.5]
Chemotherapy						
*Anthracyclines*	1 (3.1)		1 (3.8)		0 (0.0)	
*Taxanes*	7 (21.9)		5 (19.2)		5 (20.0)	
*Anthracyclines + Taxanes*	24 (75.0)		20 (77.0)		20 (80.0)	
Current endocrine therapy	24 (75.0)		19 (73.1)		20 (80.0)	
Radiotherapy	20 (62.5)		15 (57.7)		22 (88.0)	
Current psychotherapy	9 (28.1)		3 (11.5)		4 (16.0)	
Employed	25 (78.1)		20 (76.9)		20 (80.0)	
Education						
*Secondary school*	8 (25.0)		4 (15.4)		5 (20.0)	
*Higher education*	24 (75.0)		22 (84.6)		20 (80.0)	

*CI* confidence interval, *SD* standard deviation

### Working memory task performance

Accuracy or reaction time on the n-back task did not differ between groups at baseline (Table 3), so no covariates were included in the second-level model.

**Table 3:** Working memory n-back task performance at baseline

Baseline	Mean (SD)	Mindfulness	Physical training	Waitlist	*p* value
0-back	Accuracy percentage	96.08 (8.80)	95.00 (12.28)	92.97 (13.14)	.58
	Reaction time (sec)	0.50 (0.06)	0.51 (0.12)	0.51 (0.08)	.80
1-back	Accuracy percentage	84.65 (20.77)	83.20 (19.50)	84.78 (17.16)	.94
	Reaction time (sec)	0.73 (0.27)	0.71 (0.18)	0.73 (0.19)	.90
2-back	Accuracy percentage	84.72 (16.57)	78.76 (20.90)	75.96 (21.37)	.21
	Reaction time (sec)	0.82 (0.29)	0.86 (0.27)	0.81 (0.22)	.78
3-back	Accuracy percentage	65.82 (17.82)	63.85 (18.99)	66.70 (25.26)	.86
	Reaction time (sec)	0.96 (0.34)	0.94 (0.31)	0.92 (0.34)	.88

*SD* standard deviation, *sec* seconds

Within the mindfulness group, accuracy on the 3-back task improved from baseline to three-month follow-up (*β*=0.55; 95%CI=[0.16, 0.94]; *p*_FDR_=.02). Within the physical training group, accuracy (*β*=0.66; 95%CI=[0.28, 1.03]; *p*_FDR_=.002) and reaction time (*β*=−0.43; 95%CI=[−0.76, −0.10]; *p*_FDR_=.01) improved from baseline to three-month follow-up for the 2-back condition. No significant changes in performance over time were found in the waitlist group.

### Task-based functional activity

**Fig. 2:**
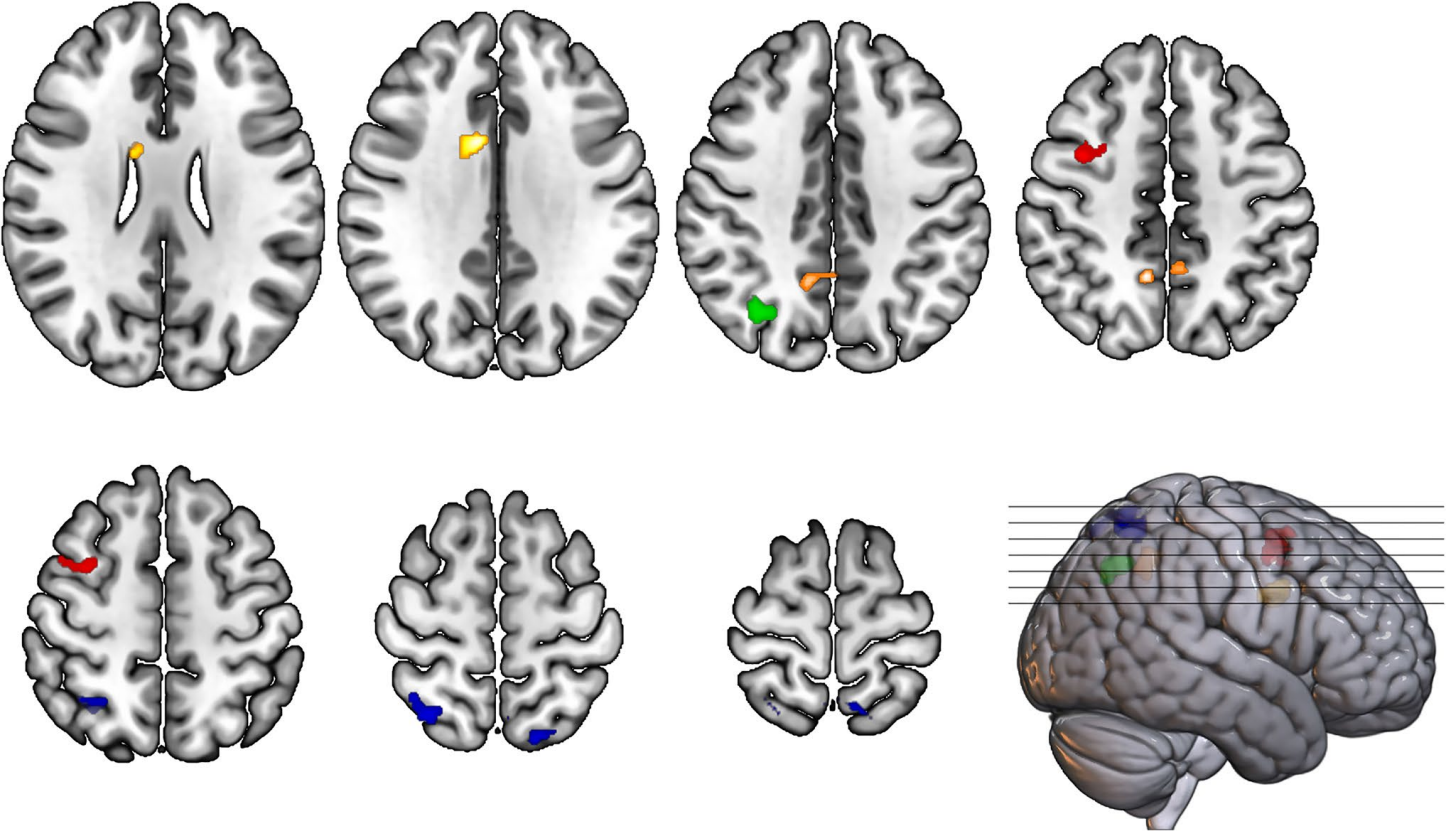
Results of between-group analysis showing areas with altered brain activation from pre- to post-intervention during a working memory n-back task. Yellow = t2<t1 and MBI>PT for 3back-0back in right dorsal anterior cingulate cortex; green = t3<t1 and MBI>WL for 2back-0back in right angular gyrus; orange = t2>t1 and MBI>WL for 3back-0back in right dorsal posterior cingulate cortex; red = t2<t1 and MBI>WL for 2back-0back in right middle frontal gyrus; blue = t3<t1 and MBI>PT for 2back-0back in bilateral superior parietal lobule t2 immediately after the intervention, t3 3-month follow-up, MBI mindfulness-based intervention, PT physical training, WL waitlist

Significant group-by-time interaction effects are visualized in Fig. 2 and detailed in Table 4. More information about the direction of the effects can be found below.

**Table 4:** Brain regions showing significant differences in working-memory related brain activation between groups over time

Region	Group-by-time interaction effects	Cluster extent [voxels]	Peak MNI coordinates [mm] x y z			*T*-value	Cluster-level p_FWE_	Potential function
2back – 0back								
Right middle frontal gyrus (MFG)	t2<t1 and MBI>WL	159	38	2	48	4.07	.01	Working memory, attentional control, emotion regulation
Right angular gyrus (AG)	t3<t1 and MBI>WL	128	34	-62	44	4.52	.03	Working memory, attentional control
Left superior parietal lobule (SPL)	t3<t1 and MBI>PT	123	-18	-72	60	5.01	.03	Attention, top-down cognitive control, working memory, motor execution
Right superior parietal lobule (SPL)	t3<t1 and MBI>PT	163	32	-60	58	4.84	.01	Attention, top-down cognitive control, working memory, motor execution
3back – 0back								
Right dorsal posterior cingulate cortex (dPCC)	t2>t1 and MBI>WL	130	10	-50	48	4.41	.03	Self-referential processing, episodic memory, and mind-wandering
Right dorsal anterior cingulate cortex (dACC)	t2<t1 and MBI>PT	142	10	8	34	5.22	.02	Error-detection, pain-, emotion-, and reward-related processing

*FWE* family wise error corrected, *MBI* mindfulness-based intervention, *PT* physical training, *t1* baseline*, t2* immediately post-intervention, *t3* three-month post-intervention, *WL* waitlist control group

#### 2back-0back working memory load

Comparing MBI participants to waitlist controls, activation decreased within the right middle frontal gyrus (MFG) in the mindfulness group (*β*=−0.49; 95%CI=[−0.80, −0.18]; *p*=.003) and increased within the waitlist group (*β*=0.76; 95%CI=[0.41, 1.11]; *p*<.001) from baseline to immediately post-intervention. From baseline to three-month follow-up, there was a non-significant reduction in brain activation within the right angular gyrus (AG) in the mindfulness group (*β*=−0.29; 95%CI=[−0.58, 0.00]; *p*=.05) and increased activation in the waitlist group (*β*=0.81; 95%CI=[0.48, 1.14]; *p*<.001).

Comparing MBI to physical training participants, we found stable brain activation in the mindfulness group (*β*=−0.16; 95%CI=[−0.39, 0.06]; *p*=.17) and increased activation in the physical training group (*β*=0.66; 95%CI=[0.41, 0.91]; *p*<.001) at three-month follow-up compared to baseline in the left superior parietal lobule (SPL). In the right SPL, activation decreased in the mindfulness group (*β*=−0.26; 95%CI=[−0.49, −0.02]; *p*=.04) and increased in the physical training group (*β*=0.62; 95%CI=[0.35, 0.88]; *p*<.001).

#### 3back-0back working memory load

Comparing MBI participants to waitlist controls, activation within the right dorsal posterior cingulate cortex (dPCC) increased within the mindfulness group (*β*=0.42; 95%CI=[0.12, 0.73]; *p*=.01) and decreased within the waitlist group (*β*=−0.76; 95%CI=[−1.11, −0.41]; *p*<.001) from baseline to immediately post-intervention.

Comparing MBI to physical training participants, activation within the right dorsal anterior cingulate cortex (dACC) decreased in the mindfulness group (*β*=−0.70; 95%CI=[−1.12, −0.29]; *p*=.001) and increased in the physical training group (*β*=1.02; 95%CI=[0.56, 1.48]; *p*<.001) from baseline to immediately post-intervention.

### Correlation with self-report questionnaires and task performance

We found a positive correlation between the change in right MFG activation and the change in scores on the Depression Anxiety Stress Scale (DASS) (t2−t1: *ρ*=.26, *p*=.02). Additionally, we found a positive correlation between the change in right SPL activation and the change in scores on the DASS (t3−t1: *ρ*=.22, *p*=.04). However, the results did not remain significant after multiple comparison correction (*p*_FDR_=.10 and .21 respectively). We found no significant correlation between task-related activation changes and changes in subjective cognitive impairment, fatigue, quality of life, or mindfulness skills.

Additionally, we found a positive correlation between the change in bilateral SPL activation and the change in accuracy percentage on the 2-back condition (t3−t1: right: *ρ*=.24, *p*=.03; left: *ρ*=.22, *p*=.04). However, the results did not remain significant after multiple comparison correction (*p*_FDR_=.09 and .05 respectively). No other significant correlations were found between brain activation and task accuracy or reaction time.

## Discussion

We investigated the impact of a MBI on working memory-related brain activation in breast cancer survivors with cognitive complaints. In line with our hypothesis, MBI showed reduced brain activation compared to no intervention or physical training in areas associated with working memory. Additionally, MBI participants showed increased brain activation in a default mode network region compared to waitlist controls. In contrast to our hypothesis, no differences in brain activation between the physical training and waitlist group could be identified.

Compared to the waitlist group, MBI participants showed reduced brain activation in the right MFG and AG. Both regions have been associated with working memory [30, 31] and attentional control [30]–[32]. As survivors showed stable performance on the working memory task immediately after the intervention, reduced activation in both brain areas might reflect a reduced need for working memory resources after MBI because cognitive resources are more efficiently allocated. Furthermore, activity in the MFG has been linked to improved emotion regulation after standardized MBIs [33, 34]. In our study, a reduction in MFG activation over time was linked to decreased self-reported emotional distress. However, these results did not survive multiple comparison correction, so we must be cautious in interpretation. Interestingly, a study in breast cancer survivors with chronic neuropathic pain showed increased gray matter volume in the MFG and AG after a standardized 8-week MBI compared to waitlist controls [35]. This provides convergent evidence for the effect of MBIs on these regions. By being aware of their experiences with an open and non-judgmental attitude, MBI participants might develop a different relationship with stressful experiences or sensations [36], including concern or worry about cognitive failures or performance, potentially leading to an increased capacity to focus on the working memory task.

Additionally, increased activation after MBI compared to waitlist controls was found in the right dPCC. The PCC is considered a key hub of the default mode network (DMN) [37], involved in self-referential processing, episodic memory, and mind-wandering [38]. The DMN directs attention to endogenous stimuli during task performance, whereas the frontoparietal network directs attention to exogeneous stimuli. Both networks thus tend to work together during the execution of a task, often showing opposite patterns of brain activation [39]. In this study, we indeed found an opposite pattern of brain activation in a region related to the DMN compared to frontoparietal network-related regions. Hence, MBI participants might have been more efficient in performing the working memory task by redirecting their attention from endogenous to exogenous stimuli more rapidly while maintaining the same task performance.

Compared to the physical training group, MBI participants showed reduced brain activation in the bilateral SPL. The SPL can be considered part of the dorsal attention network, involved in externally directed attention and top-down cognitive control [37]. Furthermore, the SPL has been linked to working memory processing [40] and motor execution [41]. In our study, reduced brain activation was associated with a decrease in self-reported emotional distress, although this finding did not survive multiple comparison correction. Hence, similar to the differences with the waitlist group, reduced activation in the SPL might reflect a reduced need for working memory resources after MBI due to improved emotion regulation and attentional control. However, the increased activation in the physical training group might not reflect an increased need for working memory resources to maintain the same performance level, as the physical training group improved on the working memory task over time. Increased task performance indeed correlated with the observed increase in brain activation, although this finding did not survive multiple comparison correction. This might suggest that physical training helps survivors to compensate more actively for working memory deficits, resulting in increased brain activation, whereas MBI might indirectly help to compensate for working memory deficits, resulting in decreased brain activation. Additionally, we observed decreased activation in the right dACC after MBI compared to physical training. The ACC is considered a key hub of the salience network [37], with a role in error-detection [42], pain-, emotion-, and reward-related processing [43, 44]. After MBI, survivors might detect errors faster and react less emotionally when making an error [45]. In our pilot study investigating breast cancer survivors with cognitive complaints, we found that increased functional connectivity between the ACC and intraparietal sulcus after MBI was associated with a reduction in emotional distress [46]. Thus, although unexpected, we believe that the difference between the MBI and physical training group might be partially explained by differences in emotion processing. More specifically, non-judgmentally attending to errors without reacting to them might increase the capacity of MBI participants to focus on the working memory task. Hence, we speculate that non-judgmental awareness and nonreactivity are two important components of MBI that might explain the differences with the physical training program. In contrast, after physical training, the functional network encompassing regions involved in attention, executive function, and emotion processing might be organized more efficiently [17]. In turn, this might aid in higher order cognitive processing to improve cognitive functions like working memory capacity. However, more research on the neural activity underlying the effects of physical training on working memory capacity is needed [47].

## Limitations and future research

While this study provides evidence for an effect of MBI on working memory related brain activation, we must consider some limitations when interpreting the findings. First, generalizability might be limited because our study population only consisted of female, Caucasian breast cancer survivors. Future studies could benefit from investigating male cancer survivors, different types of non-central nervous system cancers, and chemotherapy regimens, as well as including a more racially and ethnically diverse sample. Second, we did not collect information on baseline levels of physical activity, neither do we know whether participants in the other groups increased their physical activity behavior throughout the study. Future research could assess physical activity frequency to control for this potential confounding variable. Third, we did not find differences between the physical training and waitlist group. To date, it remains unclear which type of physical training interventions might improve cognitive functioning in breast cancer survivors [48]. Future research could adapt the physical training to individual needs, as self-selected exercise modality, intensity, and context might increase the treatment effect [49]. Finally, to further increase compliance and intervention effects, future research could assess why participants did not practice daily at home. The lack of daily home practice might be associated with CRCI-related factors like fatigue, emotional distress, or problems with planning. Adapting the interventions to the needs of cancer survivors might be essential to increase the intervention effects.

## Conclusion

After MBI, brain activation was significantly altered across brain regions involved in working memory, attentional control, and emotion processing during performance of a working memory task in a sample of breast cancer survivors with cognitive complaints. This research suggests the potential clinical utility of MBIs as a non-invasive intervention for adult women who report cognitive complaints after breast cancer treatment.

## Data Availability

The dataset generated and analyzed during the current study are not publicly available as participants did not consent to use their data outside this study.

## References

[1] Dijkshoorn ABC, van Stralen HE, Sloots M, Schagen SB, Visser-Meily JMA, Schepers VPM. Prevalence of cognitive impairment and change in patients with breast cancer: a systematic review of longitudinal studies. Psychooncology. 2021;30(5):635–48. 33533166 10.1002/pon.5623 PMC8248098

[2] Lange M, et al. Cognitive complaints in cancer survivors and expectations for support: results from a web–based survey. Cancer Med. 2019;8(5):2654. 30884207 10.1002/cam4.2069 PMC6536919

[3] Lange M, et al. Cancer-related cognitive impairment: an update on state of the art, detection, and management strategies in cancer survivors. Ann Oncol. 2019;30(12):1925–40. 31617564 10.1093/annonc/mdz410 PMC8109411

[4] Mounier NM, Abdel-Maged AES, Wahdan SA, Gad AM, Azab SS. Chemotherapy-induced cognitive impairment (CICI): an overview of etiology and pathogenesis. Life Sci. 2020;258:118071. 32673664 10.1016/j.lfs.2020.118071

[5] Feng Y, Zhang XD, Zheng G, Zhang LJ. Chemotherapy-induced brain changes in breast cancer survivors: evaluation with multimodality magnetic resonance imaging. Brain Imaging Behav. 2019;13(6):1799–814. 30937827 10.1007/s11682-019-00074-y

[6] McDonald BC, Conroy SK, Ahles TA, West JD, Saykin AJ. Alterations in brain activation during working memory processing associated with breast cancer and treatment: a prospective functional magnetic resonance imaging study. J Clin Oncol. 2012;30(20):2500–8. 22665542 10.1200/JCO.2011.38.5674 PMC3397784

[7] Wang L, et al. Reduced prefrontal activation during working and long-term memory tasks and impaired patient-reported cognition among cancer survivors postchemotherapy compared with healthy controls. Cancer. 2016;122(2):258–68. 26484435 10.1002/cncr.29737 PMC4707984

[8] Kabat-Zinn J. Full catastrophe living: using the wisdom of your body and mind to face stress, pain, and illness. New York: Bantam Books; 2013.

[9] Whitfield T, et al. The effect of mindfulness-based programs on cognitive function in adults: a systematic review and meta-analysis. Neuropsychol Rev. 2022;32(3):677–702. 10.1007/s11065-021-09519-y PMC9381612 34350544

[10] Lao SA, Kissane D, Meadows G. Cognitive effects of MBSR/MBCT: a systematic review of neuropsychological outcomes. In: Consciousness and Cognition, vol. 45. Academic Press Inc.; 2016. p. 109–23. 10.1016/j.concog.2016.08.017 27580462

[11] Jha AP, Denkova E, Zanesco AP, Witkin JE, Rooks J, Rogers SL. Does mindfulness training help working memory ‘work’ better? Curr Opin Psychol. 2019;28:273–8. 30999122 10.1016/j.copsyc.2019.02.012

[12] Weder BJ. Mindfulness in the focus of the neurosciences—the contribution of neuroimaging to the understanding of mindfulness. Front Behav Neurosci. 2022;16:310. 10.3389/fnbeh.2022.928522 PMC9622333 36325155

[13] Van Der Gucht K, et al. A mindfulness-based intervention for breast cancer patients with cognitive impairment after chemotherapy: study protocol of a three-group randomized controlled trial. Trials. 2020;21(1):1–11. 32293533 10.1186/s13063-020-4204-8 PMC7092531

[14] Ponds R, van Boxtel M, Jolles J. De ‘Cognitive Failure Questionnaire’ als maat voor subjectief cognitief functioneren. Tijdschr voor Neuropsychol. 2006;2:37–45.

[15] Melis M, et al. The impact of mindfulness on cancer-related cognitive impairment in breast cancer survivors with cognitive complaints. Cancer. 2023; 10.1002/cncr.34640 36625501

[16] Melis M, et al. Structural brain changes after a mindfulness-based intervention in breast cancer survivors with cognitive complaints. Mindfulness (N. Y). 2023;1:1–16.

[17] Melis M, et al. The impact of mindfulness on functional brain connectivity and peripheral inflammation in breast cancer survivors with cognitive complaints. Cancers (Basel). 2023;15(14):3632. 37509292 10.3390/cancers15143632 PMC10377401

[18] Kabat-Zinn J. Full catastrophe living: how to cope with stress, pain and illness using mindfulness meditation, 15th ed. Delacorte Press; 2001.

[19] Bartley T. Mindfulness-based cognitive therapy for cancer: gently turning towards. John Wiley & Sons; 2012.

[20] WHO. WHO | Physical activity and adults. WHO; 2015.

[21] Gorgolewski KJ, et al. The brain imaging data structure, a format for organizing and describing outputs of neuroimaging experiments. Sci Data. 2016;3(1):1–9. 10.1038/sdata.2016.44 PMC4978148 27326542

[22] McAllister TW, Sparling MB, Flashman LA, Guerin SJ, Mamourian AC, Saykin AJ. Differential working memory load effects after mild traumatic brain injury. Neuroimage. 2001;14(5):1004–12. 11697932 10.1006/nimg.2001.0899

[23] Bates D, Mächler M, Bolker BM, Walker SC. Fitting linear mixed-effects models using lme4. J Stat Softw. 2015;67(1):48.

[24] Lorah J. Effect size measures for multilevel models: definition, interpretation, and TIMSS example. Large-Scale Assessments Educ. 2018;6(1):1–11.

[25] Benjamini Y, Hochberg Y. Controlling the false discovery rate—a practical and powerful approach to multiple testing. J R Stat Soc Ser B Methodol. 1955;57(1):289–300.

[26] Esteban O, Birman D, Schaer M, Koyejo OO, Poldrack RA, Gorgolewski KJ. MRIQC: advancing the automatic prediction of image quality in MRI from unseen sites. PLoS One. 2017;12(9):e0184661. 28945803 10.1371/journal.pone.0184661 PMC5612458

[27] Friston KJ, Holmes AP, Worsley KJ, Poline J-P, Frith CD, Frackowiak RSJ. Statistical parametric maps in functional imaging: a general linear approach. Hum Brain Mapp. 1994;2(4):189–210.

[28] Dumas JA, et al. Chemotherapy altered brain functional connectivity in women with breast cancer: a pilot study. Brain Imaging Behav. 2013;7(4):524–32. 23852814 10.1007/s11682-013-9244-1 PMC3852152

[29] McDonald BC, et al. Multimodal MRI examination of structural and functional brain changes in older women with breast cancer in the first year of antiestrogen hormonal therapy. medRxiv. 2022:22271510. 10.1007/s10549-022-06597-1 PMC9255382 35476252

[30] Koyama MS, O’Connor D, Shehzad Z, Milham MP. Differential contributions of the middle frontal gyrus functional connectivity to literacy and numeracy. Sci Rep. 2017;7(1) 10.1038/s41598-017-17702-6 PMC5727510 29235506

[31] Seghier ML. The angular gyrus: multiple functions and multiple subdivisions. Neurosci. 2013;19(1):43. 10.1177/1073858412440596 PMC4107834 22547530

[32] Japee S, Holiday K, Satyshur MD, Mukai I, Ungerleider LG. A role of right middle frontal gyrus in reorienting of attention: a case study. Front Syst Neurosci. 2015;9:23. 25784862 10.3389/fnsys.2015.00023 PMC4347607

[33] Hölzel BK, et al. Neural mechanisms of symptom improvements in generalized anxiety disorder following mindfulness training. NeuroImage Clin. 2013;2(1):448–58. 24179799 10.1016/j.nicl.2013.03.011 PMC3777795

[34] Turpyn CC, et al. Affective neural mechanisms of a parenting-focused mindfulness intervention. Mindfulness (N. Y). 2021;12(2):392–404. 33737986 10.1007/s12671-019-01118-6 PMC7962669

[35] Hatchard T, et al. Increased gray matter following mindfulness-based stress reduction in breast cancer survivors with chronic neuropathic pain: preliminary evidence using voxel-based morphometry. Acta Neurol Belg. 2022;122(3):735–43. 35113361 10.1007/s13760-022-01877-5

[36] Bishop SR, et al. Mindfulness: a proposed operational definition. Clin Psychol Sci Pract. 2006;11(3):230–41.

[37] Spreng RN, Sepulcre J, Turner GR, Stevens WD, Schacter DL. Intrinsic architecture underlying the relations among the default, dorsal attention, and frontoparietal control networks of the human brain. J Cogn Neurosci. 2013;25(1):74–86. 22905821 10.1162/jocn_a_00281 PMC3816715

[38] Raichle ME. The brain’s default mode network. Annu Rev Neurosci. 2015;38(1):433–47. 25938726 10.1146/annurev-neuro-071013-014030

[39] Spreng N. The fallacy of a ‘task-negative’ network. Front Psychol. 2012;3:145. 22593750 10.3389/fpsyg.2012.00145 PMC3349953

[40] Koenigs M, Barbey AK, Postle BR, Grafman J. Superior parietal cortex is critical for the manipulation of information in working memory. J Neurosci. 2009;29(47):14980. 19940193 10.1523/JNEUROSCI.3706-09.2009 PMC2799248

[41] Grèzes J, Decety J. Functional anatomy of execution, mental simulation, observation, and verb generation of actions: a meta-analysis. Hum Brain Mapp. 2001;12:1–19. 11198101 10.1002/1097-0193(200101)12:1<1::AID-HBM10>3.0.CO;2-V PMC6872039

[42] Zago L, Petit L, Turbelin MR, Andersson F, Vigneau M, Tzourio-Mazoyer N. How verbal and spatial manipulation networks contribute to calculation: an fMRI study. Neuropsychologia. 2008;46(9):2403–14. 18406434 10.1016/j.neuropsychologia.2008.03.001

[43] Rolls E. T. The cingulate cortex and limbic systems for emotion, action, and memory. Brain Struct Funct. 2019;224(9):3001–18. 31451898 10.1007/s00429-019-01945-2 PMC6875144

[44] Grant JA, Courtemanche J, Duerden EG, Duncan GH, Rainville P. Cortical thickness and pain sensitivity in zen meditators. Emotion. 2010;10(1):43–53. 20141301 10.1037/a0018334

[45] Wheeler MS, Arnkoff DB, Glass CR. The neuroscience of mindfulness: how mindfulness alters the brain and facilitates emotion regulation. Mindfulness (N. Y). 2017;8(6):1471–87.

[46] Van der Gucht K, et al. Effects of a mindfulness-based intervention on cancer-related cognitive impairment: results of a randomized controlled functional magnetic resonance imaging pilot study. Cancer. 2020;126(18):4246–55. 32639592 10.1002/cncr.33074

[47] A. Wurz, G. Ayson, A. M. Smith, and J. Brunet, “A proof-of-concept sub-study exploring feasibility and preliminary evidence for the role of physical activity on neural activity during executive functioning tasks among young adults after cancer treatment.” BMC Neurol. 21(1) 2021. 10.1186/s12883-021-02280-y PMC8336393 34344355

[48] Wirtz P, Baumann FT. Physical activity, exercise and breast cancer—what is the evidence for rehabilitation, aftercare, and survival: a review. Breast Care. 2018;13(2):92–100. 10.1159/000488717 PMC5981588 29887785

[49] Zimmer P, et al. Influence of personalized exercise recommendations during rehabilitation on the sustainability of objectively measured physical activity levels, fatigue, and fatigue-related biomarkers in patients with breast cancer. Integr Cancer Ther. 2017;17(2):306–11. 28617135 10.1177/1534735417713301 PMC6041930

